# Challenges in management and prevention of ischemic heart disease in low socioeconomic status people in LLMICs

**DOI:** 10.1186/s12916-019-1454-y

**Published:** 2019-11-26

**Authors:** Rajeev Gupta, Salim Yusuf

**Affiliations:** 1 Department of Preventive Cardiology M-Floor, Eternal Heart Care Centre & Research Institute, Jawahar Circle, Jaipur, 302017 India; 2 Academic Research Development Unit, Rajasthan University of Health Sciences, Jaipur, India; 3 Population Health Research Institute, Hamilton Health Sciences and McMaster University, Hamilton, Ontario Canada

**Keywords:** Ischemic heart disease, Cardiovascular diseases, Risk factors, Acute coronary syndrome, Secondary prevention, Primary prevention

## Abstract

**Background:**

Cardiovascular diseases, principally ischemic heart disease (IHD), are the most important cause of death and disability in the majority of low- and lower-middle-income countries (LLMICs). In these countries, IHD mortality rates are significantly greater in individuals of a low socioeconomic status (SES).

**Main text:**

Three important focus areas for decreasing IHD mortality among those of low SES in LLMICs are (1) acute coronary care; (2) cardiac rehabilitation and secondary prevention; and (3) primary prevention. Greater mortality in low SES patients with acute coronary syndrome is due to lack of awareness of symptoms in patients and primary care physicians, delay in reaching healthcare facilities, non-availability of thrombolysis and coronary revascularization, and the non-affordability of expensive medicines (statins, dual anti-platelets, renin-angiotensin system blockers). Facilities for rapid diagnosis and accessible and affordable long-term care at secondary and tertiary care hospitals for IHD care are needed. A strong focus on the social determinants of health (low education, poverty, working and living conditions), greater healthcare financing, and efficient primary care is required. The quality of primary prevention needs to be improved with initiatives to eliminate tobacco and trans-fats and to reduce the consumption of alcohol, refined carbohydrates, and salt along with the promotion of healthy foods and physical activity. Efficient primary care with a focus on management of blood pressure, lipids and diabetes is needed. Task sharing with community health workers, electronic decision support systems, and use of fixed-dose combinations of blood pressure-lowering drugs and statins can substantially reduce risk factors and potentially lead to large reductions in IHD. Finally, training of physicians, nurses, and health workers in IHD prevention should be strengthened.

**Conclusion:**

The management and prevention of IHD in individuals with a low SES in LLMICs are poor. Greater availability, access, and affordability for acute coronary syndrome management and secondary prevention are important. Primary prevention should focus on tackling the social determinants of health as well as policy and individual interventions for risk factor control, supported by task sharing and use of technology.

## Background

**Fig. 1:**
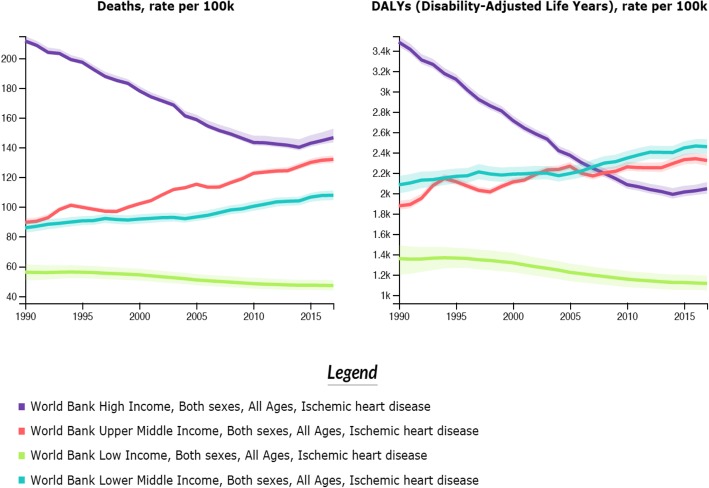
Trends (1990–2017) in ischemic heart disease mortality and burden (rates/100,000) in countries at various level of economic and social development. Based on World Bank income Categorization. Graphs plotted from data available at http://ghdx.healthdata.org/gbd-results-tool

Cardiovascular diseases (CVD), especially ischemic heart disease (IHD), are the most common causes of death and morbidity worldwide, and more than 80% of deaths occur in low- and lower-middle-income countries (LLMICs) [1]. This is due to a decline in competing causes, such as maternal, childhood, and infectious diseases, and aging of the population, along with increases in IHD risk factors, including smoking, unhealthy diet, sedentariness, hypertension, diabetes, and high blood cholesterol, in LLMICs [2]. The Global Burden of Disease study reported trends in IHD mortality in countries at various levels of socioeconomic development from 1990 to 2017 (Fig. 1). In several LLMICs, IHD mortality as well as disease burden (measured as disability adjusted life years) has increased, while these have declined in most high-income countries (HICs) [3]. Indeed, premature onset of IHD, at age less than 50 years, is especially important in LLMICs [4].

**Fig. 2:**
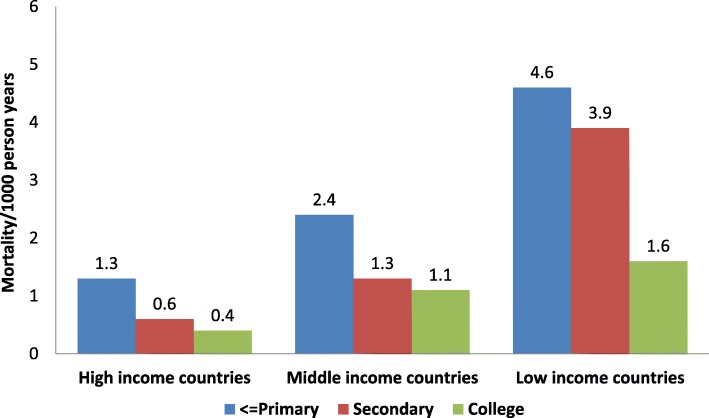
Educational status categories (≤ primary, secondary and college) and age- and sex-standardized cardiovascular mortality in high-income, middle-income and low-income countries in the Prospective Urban Rural Epidemiology (PURE) study (21 countries, *n* = 160,299) [6]

The reduction in the burden of IHD in HICs and upper-middle-income countries in the past 50 years has been achieved through strategies involving better management of acute and chronic IHD as well as its primary prevention [2]. The Prospective Urban Rural Epidemiology (PURE) study reported that, in LLMICs, there is an IHD paradox characterized by greater mortality despite lower burden of CVD risk factors compared to HICs and upper-middle-income countries, where risk factors are higher and disease incidence and mortality are lower [5]. The PURE study also reported that cardiovascular mortality was significantly greater (almost threefold) in individuals of a low socioeconomic status (SES) in HICs, middle-income (MICs) and low-income countries (LICs) [6]. Mortality was the highest in those of low SES in LICs (Fig. 2) despite the lower prevalence of risk factors (INTERHEART risk score) [6]; this paradox could be due to the inferior quality of acute and chronic IHD management and poor risk factor control [7].

IHD prevention involves primordial, primary, and secondary prevention [8]. Primordial prevention is defined as preventing the onset of the risk factors by addressing the underlying political, social, and economic determinants at the population level [9]. Primary prevention involves the control of major cardiovascular risk factors (tobacco use, high blood pressure (BP), cholesterol, diabetes, etc.) among individuals identified through systematic or opportunistic screening. Modeling studies in Europe and the USA have reported that 50–60% of the decline in IHD mortality is attributable to prevention strategies at both population and individual levels [10, 11]. High quality acute coronary disease management and secondary prevention for those who have survived the initial coronary event are important and responsible for 30–40% of the IHD mortality decline in HICs [11]. However, prevention efforts have shown variable results in different countries. In Finland, the effects of primary prevention seem to dominate, yet in several other MICs and upper-middle-income countries in Europe, Americas and Asia, the decline is due to improved clinical management and secondary prevention, as reported in MONICA cohorts [12]. We believe that improving acute coronary syndrome care and better secondary prevention can significantly reduce IHD mortality in LLMICs [13]. Herein, we initially focus on gaps in the quality of acute IHD management and secondary prevention and then highlight the importance of primary prevention in LLMICs, especially among the more vulnerable individuals of lower SES. We also highlight a few strategies to overcome these challenges.

## Acute coronary syndrome management

Over the past decades, acute coronary syndrome (ACS) management has improved significantly following technological and pharmaceutical innovations that have led to improved pre-hospital diagnosis and treatments, more rapid admission to hospital, greater use of proven therapies delivered in coronary care units, increased use of defibrillators, pace-makers and acute percutaneous coronary interventions, drugs (anti-platelets, heparin, thrombolytics, beta-blockers, angiotensin-converting enzyme inhibitors and statins), and appropriate cardiac rehabilitation [13]. Many of these therapies are underused in most LLMICs [13–15], especially among those of low SES [7, 14]. The factors of importance leading to greater mortality in individuals of low SES in LLMICs include the poor access and availability of lifesaving therapies as well as a low quality of care [7, 14–16]. The implementation of guideline-based management of ACS using validated protocols could facilitate better management.

### Access to acute IHD care

Access to high-quality care for ACS is an important impediment for IHD mortality reduction in those of low SES in most LLMICs. There are only limited data on population-based ACS registries in LLMICs. In India, for example, the Million Death Study investigators reported that more than three-quarters of deaths from CVD occur at home, and significantly more in rural than in urban populations, suggesting the non-availability of care or a failure to access care [17]. The PURE study reported that IHD mortality was significantly greater in rural participants than among people in urban communities in LICs [5, 18]. ACS registries from LLMICs have reported delays in diagnosis due to diversion to a primary care practitioner, which delays admission to a hospital within the required time for various evidence-based treatments; such delays are more common among those of low SES [19]. Additionally, in those of lower SES, there is also a lack of awareness of symptoms, sparse availability of primary care, absence of ambulances (patients use own or rented transportation), poor availability of diagnostic services at primary care clinics (electrocardiogram, etc.), out-of-pocket expenses for expensive medicines and coronary interventions, and substantial delays in obtaining insurance approvals [20]. Furthermore, catastrophic health expenses are common in those of low SES in LLMICs [21].

Some policy initiatives have been implemented in many LLMICs to provide rapid access to high quality ACS care such as, for example, efforts to provide free ambulance services for emergencies, the creation of systems for central telediagnosis and telemonitoring, and rapid transfer of patients to facilities with capabilities for pharmacological reperfusion or coronary interventions for underserved populations in Africa, Latin America, and India [16, 22, 23]. Pilot projects on pre-hospital thrombolysis using nurse-practitioners or primary care physicians are being evaluated in some LLMICs [24, 25]. However, high-quality randomized clinical trials and economic evaluation of technology-supported interventions are not yet available [26]. Furthermore, poverty alleviation and improving health literacy among the general population and heart-literacy among primary care nurses and physicians are important for symptom identification and rapid transport of patients for ACS management [27]. These efforts involve attempts to improve health literacy, provide access, and task-shifting strategies whereby some simple but critical tasks are shifted from physicians to trained non-physicians for risk identification, risk management, and early diagnosis [28–30].

### Quality of care

**Fig. 3:**
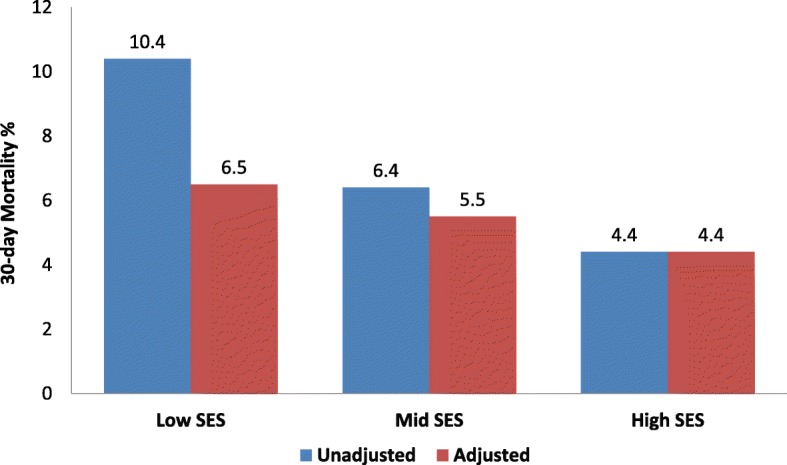
Thirty-day mortality following acute coronary syndrome according to socioeconomic status (SES) in the CREATE Registry (*n* = 20,468) in India. Significantly greater mortality is observed in the low-SES compared to mid- and high-SES patients. The difference is attenuated after adjustment for interventions, reperfusion therapies, other evidence-based therapies, and risk factors [20]

ACS registries from India and LLMICs have reported that 30-day mortality is significantly greater in these countries as compared to registries in Europe and the USA [7]. These registries have also reported lower use of thrombolytics and other reperfusion strategies, renin–angiotensin system (RAS) blockers, statins, and beta-blockers in ACS patients. Within LLMICs, ACS patients of low SES have significantly greater in-hospital and 30-day mortality [31]. The CREATE Registry, included 20,468 patients with ACS from multiple sites in India [20], reported significantly greater 30-day mortality in individuals of low and middle SES compared to those of high SES (10.4% vs. 6.4 and 4.4%, respectively). The differences persisted after adjustment of risk factors but were significantly attenuated after adjustment for differences in the rates of use of various evidence-based therapies (Fig. 3). Similar data have been reported in more recent registries in LLMICs [31].

Several strategies to improve the quality of care have been tried in LLMICs, including greater financing for the creation of infrastructure and support medical personnel, creation of coronary care units at secondary level hospitals, invasive cardiology centers with 24 × 7 availability of interventional cardiologists, and health insurance to increase the affordability of care [14, 16, 31]. Other strategies include telemedicine-enabled diagnosis and algorithm-based management, better training of physicians for improving ACS care, and use of discharge checklists to ensure that proven secondary prevention strategies have been implemented [31, 32]. Publicly funded insurance schemes and free medicine supply schemes have also been implemented. However, the results of most of the initiatives have been equivocal and no study has reported clear reductions in clinical outcomes [32], perhaps because the changes in rates of use of key therapies were modest.

## Cardiac rehabilitation and secondary prevention

High quality cardiac rehabilitation and secondary prevention are associated with a decreased incidence of post-discharge coronary events and save lives [33, 34]. An overview of 6 Cochrane systematic reviews concluded that exercise-based cardiac rehabilitation decreased hospital admissions and improved health-related quality of life compared to usual care and could reduce mortality in the long term [35]. In another meta-analysis of 29 studies, a 26% lower (confidence intervals 14–36%) risk of cardiovascular mortality was reported [36]. Nevertheless, there are only limited cardiac rehabilitation programs in LLMICs, none of which involve individuals of low SES [34]; the few that exist are provided by private hospitals, which are too expensive for the average individual. Moreover, the impact of these programs on outcomes has not been reported.

**Fig. 4:**
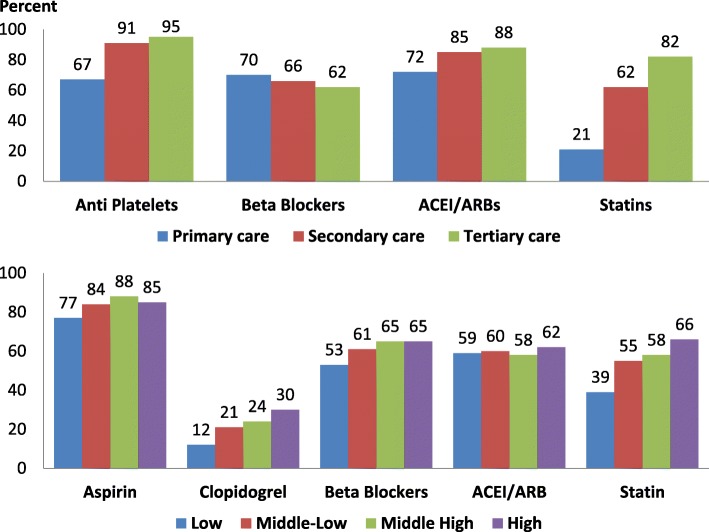
Prescription audit in India (*n* = 2993) shows significantly lower use of statins, angiotensin-converting enzyme inhibitors/angiotensin receptor blockers and anti-platelet drugs in stable ischemic heart disease patients at primary care (low socioeconomic status) compared to secondary and tertiary care clinics (upper graph) [43]. Similar results have been reported among low educational status patients from China in a nationally representative cohort (*n* = 2803) (lower graph) [44]

The secondary prevention strategy focuses on promoting evidence-based drug therapies. Reviews have reported incremental benefit of post-discharge drug therapies – concurrent therapies with aspirin, beta-blockers, angiotensin-converting enzyme inhibitors, and statins are associated with a decline of 2-year mortality from 8 to 2% following ACS [37]. The EUROASPIRE study, performed in multiple European countries, reported that countries with a lower human development index had a significantly lower adherence to healthy lifestyles (smoking cessation, physical activity, healthy diet) and secondary preventive cardiac medicines (anti-platelets, beta-blockers, RAS blockers, and statins) than those with a higher human development index [38]. The WHO-PREMISE study in 10 MICs and LLMICs reported similar results, with low adherence to drug therapies – particularly RAS blockers and statins – in LLMICs compared to MICs [39]. These studies did not report whether there were differences among people of lower SES compared to those of high SES in LLMICs. In HICs, it has been reported that patients of lower SES have less access to cardiac rehabilitation and lower adherence to healthy lifestyles and secondary prevention drug therapies [40]. In the PURE study, a very low rate of use of all cardioprotective therapies was reported in patients with known IHD and stroke in LLMICs compared to those in MICs and HICs [41]. In the South Asian cohort of the PURE study, it was reported that patients of low SES (low educational status or low wealth index) with IHD or stroke had the lowest consumption of various evidence-based therapies at approximately 4 years after diagnosis [42]. A prescription audit in India reported lower secondary prevention therapies in primary care clinics, catering to patients of low SES, compared to IHD patients in secondary and tertiary care (Fig. 4, upper panel) [43]. In China, a prescription audit among stable IHD patients from a nationally representative sample reported that a low SES was independently associated with lower rates of use of aspirin, clopidogrel, beta-blockers, and statins [44]. The treatment rates with various drugs in different educational status groups are shown in Fig. 4 (lower panel).

There are multiple reasons for the lower quality of long-term care in these countries (Table 1). Important barriers in LLMICs are at healthcare system level (availability, access and affordability of medications), healthcare provider level (quality of medical education, physician shortage, physician inertia, and lack of task-sharing), and patient level (health illiteracy, poverty, and drug costs) [31]. Conversely, important strategies that have been shown to enhance cardiac rehabilitation and secondary prevention services in LLMICs are the creation of infrastructure, universal health coverage, free medicines, physician empowerment, task-sharing with nurses and community health workers, patient and family education, and family participation in care [31, 45]. Many of these strategies have been evaluated in clinical trials, with multicomponent interventions being the most effective [31].

**Table 1:** Barriers and facilitators to lifestyle and medication adherence for secondary prevention

	Barriers	Facilitators
Healthcare system	– Low funding for non-communicable diseases – Poor access and availability of healthcare – Uninsured out-patient management – Low quality medical education	– Improvement in healthcare systems related to access, affordability, convenience – Better medical education – Involvement of non-medical professionals in healthcare – Multisectoral interventions
Healthcare providers	– Lack of understanding of patient needs – Neglect to involve patients – Poor focus on lifestyle changes – Prescribing complex regimens – Failure to explain benefits and side effects – Lack of continuity of care – Inappropriate treatment or over-treatment	– Simplifying the medication regimen, combinations, fixed dose combinations, and polypills – Improving patient education, motivation, cost awareness – Elimination of treatment inertia – Training existing community health workers, nurses, and pharmacists – Continual monitoring of patient compliance by physician or other healthcare workers – Assurance of continuity of care
Patient related	– Social isolation, especially in the elderly – Lack of motivation and commitment – Failure to realize seriousness of problem – Failure to sustain lifestyle changes – Multiple stakeholders and messages – Lack of quality information – Ancillary and drug costs – Universal healthcare and insurance cover	– Patient education and counseling – Self-monitoring of adherence to lifestyles and pharmacotherapy using technology – Behavioral strategies, e.g., self-monitoring of blood pressure and glucose, diaries, memory cues, rewards – Social support by family, health workers, physicians

## Primary prevention

Population-based prevention strategies influence many proximate coronary risk factors, including air pollution, tobacco use, consumption of unhealthy foods, sedentariness, psychosocial stress, and obesity, while primary prevention addresses risks through lifestyle changes and appropriate drug therapies [8]. Multi-level approaches at a population and individual level have been developed for primary prevention, though mostly in HICs and MICs, with only a few in LLMICs [9, 46]. Selected strategies that would produce the maximum benefit in LLMICs, especially in those of low SES, are summarized here.

### Social determinants of health

The World Health Organization (WHO) Commission on Social Determinants of Health has recommended actions to improve daily living conditions, to tackle the inequitable distribution of power, money and resources, and to measure and understand the problem and assess the impact of action for chronic disease prevention [47]. The United Nations has promulgated 17 Sustainable Development Goals to address multiple social issues (Table 2) [48], each of which has the potential to promote health, although more research is needed [49, 50]. Especially important for cardiovascular health are goals aiming to eliminate poverty, to provide good health and wellbeing, quality education, affordable and clean energy, decent working conditions and economic growth, to support innovation, and to forge action for a healthy climate [8].

**Table 2:** United Nations Sustainable Development Goals (SDGs) and health

SDG number	SDG domain	World Health Organization response
1	No poverty	Prioritizing the health needs of the poor
2	Zero hunger	Addressing the causes and consequences of all forms of malnutrition
3	Good health and wellbeing	Ensure healthy lives and promote wellbeing for all at all ages
4	Quality education	Supporting high quality education for all to improve health and health equity
5	Gender equality	Fighting gender inequality, including violence against women
6	Clean water and sanitation	Preventing disease through safe water and sanitation for all
7	Affordable and clean energy	Promoting sustainable energy for healthy homes and lives
8	Decent work and economic growth	Promoting health employment as a driver of inclusive economic growth
9	Industry, innovation, and infrastructure	Promoting national research and development capacity and manufacturing of affordable essential medical products
10	Reduced inequalities	Ensuring equitable access to health services through universal health coverage based on strong primary care
11	Sustainable cities and communities	Fostering healthier cities through urban planning and cleaner air and safer and more active living
12	Responsible consumption and production	Promoting responsible consumption of medicines to combat antibiotic resistance (or overmedication)
13	Climate action	Protective health from climate risks and promoting health through low-carbon development
14	Life below water	Supporting the restoration of fish stocks to improve safe and diversified healthy diets
15	Life on land	Promoting health and preventing disease through healthy natural environments
16	Peace, justice, and strong institutions	Empowering strong local institutions to develop, implement, monitor and account for ambitious SDG responses
17	Partnerships for the goals	Mobilizing partners to monitor and attain health-related SDGs

Low educational status is one of the most important cardiovascular risk factors in LLMICs as recently reported in the PURE study [51]. Other, observational studies have also reported an inverse gradient in CVD mortality with better education in LLMICs [52, 53]. Policies to provide universal basic education are present in most LLMICs yet, unless there is a focus on quality education (Table 2), IHD will continue to be high and an important cause of death in populations of lower SES.

A health-in-all-policies approach has also been suggested by WHO as a strategy to achieve better health [54]. This approach is focused on public policies across sectors (involving the ministries of health, education, finance, agriculture, environment, urban and rural development, human and social development, and industries [8, 54]) and systematically considers the health implications of policy decisions, seeks synergies, and avoids harmful health impacts in order to improve population health and health equity [54]. A model for entrusting coordination and implementation of policies to the national planning commission has been suggested, and some countries are now implementing this approach [28], with particular success in Finland; however, whether it can be translated to LLMICs to help those of low SES awaits further studies.

### Lifestyle risk factors

Two sets of IHD risk factors are important in LLMICs. The first relates to lifestyle factors, including smoking and other forms of tobacco use, alcohol abuse, poor quality diet (consumption of low quantities of fruit and vegetables and high consumption of carbohydrates, trans fats, and foods laced with chemical pollutants), indoor and ambient air pollution, and sedentariness [8]. All these risk factors are widely prevalent in LLMICs, especially among those of low SES [51]. Legislations exist to control these factors, yet the level of implementation is low. For example, although most LLMICs are signatories to the Framework Convention for Tobacco Control, fewer than 50% have taken steps to implement the recommendations [55]. Food policies are essential to curb the intake of high carbohydrate foods (by making alternative and healthier foods more available and affordable) and trans fats (through legislation) but these either do not exist or are poorly implemented [56]. Important strategies for pollution control are the publicity of their adverse effects on health, shifting to cleaner fuels (from solid fuels to cleaner alternatives such as gas and electricity for cooking), decreased use of fossil fuels for transportation and electricity generation, emission trading programs, transportation reforms, reduction in traffic emissions, and urban landscape reform [57]. Legal enforcement backed by technology, targets, and timetables are important to ensure the implementation of various policies to protect people of low SES.

### Cardiometabolic risk factors

The second set of factors emerging in LLMICs are cardiometabolic risk factors driven by increasing generalized and abdominal obesity [58]. Overweight and obesity are the modern epidemics in LLMICs [59]. The Non-Communicable Diseases Risk Factor Collaboration has reported that increasing body mass index among rural populations worldwide has narrowed the differences in body mass index between urban and rural communities [59]. Population and individual level strategies outlined in the Lancet Commission Report on Global Syndemics of Obesity, Under-nutrition and Climate Change are important [60]. Other risk factors associated with the epidemiological and food transition in LLMICs among the poor are hypertension, type 2 diabetes, hypercholesterolemia, and hypertriglyceridemia [61]. The WHO Global Status Report on Non-communicable Diseases has reported that hypertension prevalence is high in sub-Saharan Africa, South Asia, and East Asia, while diabetes is epidemic in South, East, and West Asia [61]. Additionally, hypercholesterolemia is widely prevalent in many LLMICs [62]. The PURE study has reported that hypertension is the most important risk factor for incident cardiovascular diseases in LLMICs in populations of high and low SES [6, 51].

The status of awareness, treatment, and control of these risk factors is low in most LLMICs. Hypertension control, which is a marker of overall IHD risk factor control, is very low in LLMICs [63]. Geldsetzer et al. [64] evaluated levels of hypertension control in 44 LMICs with data from 1.1 million participants and reported hypertension in 17.6%. In those with hypertension, 73.6% had had their BP measured, 39.2% were aware of their diagnosis, 29.9% received treatment, and 10.3% had it under control. However, certain LLMICs – Costa Rica, Kyrgyzstan and Bangladesh – performed better, attributed to more efficient primary healthcare, better community health worker infrastructure, and a wider availability and affordability of anti-hypertensive medications. In LLMICs, older age, female sex, non-smokers, and greater education and income were associated with improved BP control. Low rates of diabetes control and use of statins in LLMICs have also been reported [65, 66]. A population-based study in India reported rates of 10, 7, and 5%, respectively, in hypercholesterolemia awareness, treatment, and control among urban populations [67].

Strategies to improve the control of multiple IHD risk factors are required. Universal and efficient primary healthcare with a focus on cardiovascular disease primary prevention (health education, risk factor screening, appropriate lifestyle interventions and treatments) can lead to changes in health behaviors in individuals and communities [8]. Studies have reported that countries in the highest quintile of universal health coverage have lower smoking and tobacco use, BP, and hyperglycemia, all of which are evidence of better risk factor control [68]. System-wide interventional studies are needed to clearly identify the type of healthcare systems and healthcare financing models for CVD prevention. Other strategies involve educating physicians, other health workers, task-shifting or task-sharing between physicians and nurses or other health workers, and the use of digital and pharmaceutical technologies.

### Education

An important strategy for IHD prevention in LLMICs is improvement in the quality of medical education for all healthcare professionals, especially physicians, nurses, and allied health workers [8]. Suggested educational strategies involve the reorientation of undergraduate and postgraduate education with a focus on healthy lifestyles. There is a need for integrating formal learning with practical, heuristic, inquiry-driven, inter-professional, and population health management activities. It has been argued that better physician education and an enhancement of collaborative care delivery can reduce the health and economic burdens from IHD to a degree not previously realized [69]. The WHO has suggested that physicians should be adequately trained to have the proficiency to meet the demands of healthcare systems and the health needs of people while maintaining the systems needed to provide medical care to the sick [70]. It has also charged medical schools to produce graduates who are proficient to deliver preventive, promotive, curative, and rehabilitative care, especially in LLMICs [70].

### Health workers

In LLMICs, there is need for training of nurses and other non-physician health workers in the assessment and management of hypertension, lipids, tobacco use, and diabetes. Task sharing with pharmacists for hypertension management has significantly improved adherence to lifestyles and medications in MICs and HICs [71]. However, whether pharmacist- or nurse-based models can be replicated in LLMICs awaits further studies [72]. Education of community health workers for prevention is important [73]. In LLMICs, where physician shortage is widespread, task-sharing strategies with health workers in public education, lifestyle improvement, and medication adherence can lead to better control of risk factors [72]. Studies utilizing community health worker-based interventions to control cardiovascular risk factors in LLMICs have produced equivocal results [29, 73, 74]. A more intensive intervention was used in the Heart Outcomes Prevention Evaluation (HOPE)-4 study, which evaluated a multipronged strategy with non-physician health worker-led detection, treatment, and control of cardiovascular risk factors with a computer-based decision support system and polypill strategy [75]. A significant reduction of systolic BP and low-density lipoprotein cholesterol in the intervention groups has been reported [76].

### Technology

Electronic technologies (e.g., mHealth, eHealth) have the potential to provide low cost preventative interventions for cardiovascular risk reduction in LLMICs [26]. Several studies have evaluated the efficacy and effectiveness of such technologies for risk identification and diagnosis, decision support system-based management, and improving adherence to healthy lifestyle and medications using telemedicine, web-based strategies, email, mobile phones, mobile applications, text messaging, and monitoring sensors [77]. However, outcomes have been equivocal and a Cochrane review concluded that the inconsistency in quality of reporting of digital health interventions for cardiometabolic outcomes might be an impediment to real-world implementation [78]. Cost-effectiveness studies of outcome trials are required before these strategies are widely adopted in LLMICs, especially among the low SES populations.

Pharmaceutical innovations are also important. Knowledge translation of existing interventions into practice is crucial for IHD prevention in LLMICs [9]. A combination of various cardiovascular risk reduction drugs (anti-hypertensive, cholesterol lowering, and anti-platelet) into a single pill (polypill) has the potential to simplify risk management in LLMICs cost-effectively [79]. Trials using such combinations have led to significant reductions in BP and low-density lipoprotein cholesterol levels in intermediate- and high-risk individuals in India [80] and in individuals of low SES in the USA [81]. The PolyIran study is one of the first outcome studies of combination pharmacotherapy for the primary and secondary prevention of IHD [82]. In this cluster-randomized trial use of a single pill containing aspirin, atorvastatin, hydrochlorothiazide, and either enalapril or valsartan in 120 intervention clusters (3421 participants) compared to 116 minimal care clusters (3417 participants) over a 60-month follow up was associated with a 34% relative risk reduction in major cardiovascular events (95% confidence intervals 20–45%) in both primary and secondary prevention groups. It was concluded that a polypill strategy could be considered as an additional effective component in controlling CVD in LLMICs. Outcomes of ongoing studies of polypill strategies [83] along with economic evaluations will be important to confirm these findings before this strategy is widely adopted among individuals of low SES in LLMICs.

## Conclusions

Prospective data from 21 HICs, MICs and LICs in the PURE study has shown that age- and sex-standardized cardiovascular mortality is more than threefold higher in individuals of low SES in LLMICs compared to in HICs [6]. Studies have also reported that mortality from ACS in LLMICs is almost twice that in HICs and is significantly greater in rural persons of low SES [5, 6]; these differences could be due to inferior quality of care received by patients of low SES [7]. Three important focus areas for decreasing IHD mortality in LLMICs are acute coronary care, secondary prevention, and primary prevention. With regards to patients of low SES with ACS there is a lack of awareness of symptoms by both patients and primary care physicians, delays in reaching healthcare facilities, non-availability of thrombolysis and coronary revascularization, and poor affordability for medicines. Facilities for rapid diagnosis and accessible and affordable care at secondary and tertiary care hospitals for acute coronary care are needed. Similarly, facilities for cardiac rehabilitation and adherence to long-term secondary prevention therapies are sub-optimal due to poor availability, access, affordability, and physician knowledge, and must be improved. Task-sharing of physicians with community health workers could be important to promote adherence in secondary prevention. The quality of primary prevention needs to be improved with policy initiatives to control tobacco, trans-fats, refined carbohydrates, and excessive salt consumption along with the promotion of healthy foods and physical activity. Furthermore, efficient primary care with a focus on BP, lipids and tobacco control is needed [8]. Task sharing of physicians with community health workers, utilizing novel strategies for risk factor control, are required. Medical education of physicians, nurses, and health workers should be strengthened, along with similar approaches in educating patients and their families. Finally, a focus on the social determinants of health, such as education and better healthcare financing using health-in-all-policies approach, are also important.

## Data Availability

Not applicable.

## Abbreviations

ACS: Acute coronary syndrome
BP: Blood pressure
CVD: Cardiovascular diseases
HICs: High-income countries
IHD: Ischemic heart disease
LICs: Low-income countries
LLMICs: Low- and lower-middle-income countries
MICs: Middle-income countries
PURE: Prospective Urban Rural Epidemiology
RAS: Renin–angiotensin system
SES: Socioeconomic status
WHO: World Health Organization
